# Factors associated with full vaccination and zero vaccine dose in children aged 12–59 months in 6 health districts of Cameroon

**DOI:** 10.1186/s12889-023-16609-4

**Published:** 2023-09-01

**Authors:** Martin Ndinakie Yakum, Funwie Desmond Atanga, Atem Bethel Ajong, Linda Evans Eba Ze, Zahir Shah

**Affiliations:** 1 Department of Epidemiology and Biostatistics, School of Medical and Health Sciences, Kesmonds International University, Goundere, Cameroon; 2 Department of Biochemistry, Faculty of Science, University of Dschang-Cameroon, Dschang, Cameroon; 3 Department of Public Health, Faculty of Medicine and Pharmaceutical Sciences, University of Dschang-Cameroon, Dschang, Cameroon

**Keywords:** Vaccination, Zero-dose, Immunisation, Determinant, Wealth-index, Coverage, Full immunisation, Children

## Abstract

**Background:**

Routine immunisation coverage in Cameroon is still below the target of the national Expanded Programme on Immunisation (EPI), with only 42% of children fully immunised according to Demographic and Health Survey (DHS) report in 2018. The objective of this study was to evaluate factors associated with full immunisation and zero-dose in Cameroonian children.

**Methods:**

A two-stage cross-sectional cluster survey was conducted in Yaoundé in November 2021, targeting children aged 12–59 months. The clusters were chosen with probability proportionate to population size (PPS), and households selected by restricted sampling technique. Data were collected from the vaccination card of the child or from parents’ recall, if the card was not available, using electronic forms with tablets. Using R (version 4.1.0.), the proportion of fully immunised children was calculated. The household wealth index was described using principal component analysis, and factors associated with full immunisation assessed with multiple logistics regression. The threshold of statistical significance was set at 5%.

**Findings:**

A total, 273 children aged 12–59 months enrolled; 37% of participants were fully immunised, and 16% had never received any vaccine. Mother’s level of education: Primary (OR = 3.59, *p* = 0.0200), high school (OR = 3.68, *p* = 0.0400*), and higher education (OR = 8.25, *p* = 0.0018), and sharing household with biological father (OR = 2.11, *p* = 0.0305) were significantly associated with full vaccination. Living in a richer (3^rd^-5^th^ wealth quintiles) household (OR = 0.25, *p* = 0.0053); mother’s education: Primary (OR = 0.07, *p* = 0.0271) and Higher education (OR = 0.10, *p* = 0.0419), living with the mother (OR = 0.05, *p* =  < 0.0001) and living with the father (OR = 0.22, *p* = 0.0253) had significant negative association with zero-dose in children.

**Conclusion:**

The proportion of fully vaccinated children in Yaounde is lower than the national average. Children from poor homes and those borne by uneducated mother have higher odds of not being vaccinated. Immunisation programmes in Yaounde need to be stepped up to improve coverage. Equally, there is a need to reconsider how the poor can the better reached with immunisation services.

## Introduction

The effectiveness of routine immunisation programmes in reducing morbidity and mortality is well known and documented globally. A study in USA (United States of America), reported that among 78.6 million children born during 1994–2013, routine childhood immunisation was estimated to prevent 322 million illnesses and 21 million hospitalizations over the course of their lifetimes and avert 732,000 premature deaths from vaccine-preventable illnesses [1]. Thanks to immunisation, smallpox [2] has been eradicated globally and poliovirus eliminated in most WHO (World Health Organization) regions [3, 4]. In Cameroon, there has been an enormous drop in the incidence of vaccine-preventable diseases from 1980 to 2019 [5]. Despite unambiguous evidence of effectiveness, vaccination coverage remains below the desired level in several countries, including both developed and developing countries [6–8].

Despite the fact that vaccination is unquestionably the most effective public health preventive measure globally, routine vaccination coverage has dropped recently. Global estimates of vaccination coverage with 3 doses of diphtheria-tetanus-pertussis–containing vaccine (DTPcv3) decreased from an average of 86% during 2015–2019 to 83% in 2020 and 81% in 2021 [9]. In Cameroon, the full vaccination coverage in children aged 12–23 months increased slowly from 40% in 1991 to 52% in 2018 [10]. However, in comparison to the coverage in 2011, the full vaccination coverage has dropped by 1 unit in 2018 while the proportion of children aged 12–23 months who did not receive any vaccine dose doubled from 5% in 2011 to 10% in 2018 [10]. Little is known about factors associated with low vaccination coverage in Cameroon.

Cameroon has a district health system organised in three levels: the central level is represented by the ministry of public health; intermediate level is represented by the 10 regional delegations of public health; and the operational level represented by 189 health districts [11]. Each health district is made up of several health areas, hosting one or more health facilities which can be public or private facilities. Health facilities in Cameroon are classified into 7 different categories starting from the integrated health offering the basic of healthcare services to General hospital offering more specialised health services [11]. Immunisation service delivery is coordinated at the district level by the district health services office with all health facilities irrespective of their categories acting as points of immunisation service delivery [11]. The national guidelines for Expanded Programme on Immunisation (EPI) recommends that immunisation services be delivered at health facilities everyday [11]. For population living more that 5 km away from the nearest health facility or having physical barriers to reach the facility within a 1 h walk, outreach immunisation sessions are scheduled to vaccinate children on monthly basis [11]. The routine EPI in Cameroon aims to prevent 13 infectious diseases in children aged 0–11 months, with 16 vaccine doses for all children to receive from birth to 9 months of age [11]. The aims of the EPI in Cameroon is to attain coverage of at least 90% nationwide, with each district achieving a coverage of at least 80% per vaccine [12].

Little is known about the reasons for the low vaccination coverage in Cameroon. However, a study reported that vaccine hesitancy associated with routine EPI vaccine in Yaounde is estimated at 26% [13]. Other studies reported that vaccination coverage can be associated with socio-demographic and economic characteristics of the populations such as the level of education of the mother [6], the presence of the vaccination card [14], and household Wealth level [15–17]. However, only a few studies have evaluated factors associated with full immunisation and no one assessed factors associated with zero-dose in children in Cameroon. We believe that having knowledge on the context specific factors can help immunisation managers to know how to reduce immunisation dropout rate and improve immunisation uptake. The objective of this paper was to identify factors associated with full immunisation and zero vaccine dose in children aged 12–59 months in Yaounde-Cameroon.

## Materials and methods

### Research design

It was a two-stage cross-sectional survey, conducted in Yaounde-Cameroon, in November 2021. It targeted children aged 12–59 months in the community. Clusters were formed from existing neighbourhoods, and cluster selection was done by systematic sampling with probability proportionate to population size (PPS). Households were selected by a restricted sampling technique. Data collection was done by checking the routine immunisation card or by parent recall (when the card was not available). Data were cleaned using MS-Excel 2019 and analysed with R version 4.1.0 (2021–05-18).

### Research area

The study area included 6 health districts: Biyem assi, Cite verte, Djoungolo, Efoulan, Nkolbisson, and Nkolndongo. Though some of these districts have remote health areas, they are predominantly urban areas. The estimated population for the study area is 3 million inhabitants, with a total of 57 health areas and 366 health facilities. This study area was selected in order to have a picture of immunisation uptake in urban health districts as most previous studies were conducted in remotes areas except for the DHS, which included both remote and urban areas.

### Study population

This study targeted children aged 12–59 months. The EPI in Cameroon aims to fully immunise all children by their first birthday (12 months). We aimed to enrol children who were supposed to have completed their routine immunisation but we limited our target to children 59 months old because children under five years are most affected by vaccine-preventable diseases and also to reduce recall bias.

### Sample size calculation

The required sample size was estimated to be 286 children aged 12–59 months. This was estimated using the following: full immunisation coverage among children in Cameroon (*p* = 42%) [10], desired precision (d = 7%), design effect(*deff* = 1.5), and 95% confidence level(z_α/2_ = 1.96). The sample size was estimated using the single proportion formula for sample size as follows:

$$n=\frac{{{Z}_{\frac{\alpha }{2}}}^{2}*p*\left(1-p\right)*deff}{{d}^{2}}$$

### Sampling methods

Firstly, an exhaustive list of clusters (formed from existing quarters in the study area) was prepared to form the sampling frame. A total of 30 clusters were then selected using systematic sampling with probability proportionate to population size (PPS), using the ENA (Emergency Nutrition Assessment) software version 2021. Secondly, 24 households were randomly selected from each cluster using a modified systematic sampling in which one household was randomly selected at successive “sampling interval”. We prefer to call this sampling method “restricted sampling”. To better explain this method, the “sampling interval” was calculated as in systematic sampling, but we call it the block size, and we randomly selected one household within each block until we got the required number of households. All children aged 12–59 months in a selected household were included in the study. In the restricted sampling technique, the selection of a household is not determined by the previously interviewed household, as it is the case with systematic sampling. We believe this method gives more room to chance in the selection.

### Data collection

The questionnaire used in this study was derived from the immunisation coverage survey questionnaire used by the DHS in Cameroon, in 2018. Data collectors were trained, research tools pretested, and data collection was closely supervised in the field. Data were collected using an electronic questionnaire designed in KoBo Toolbox.

Data on the immunisation status of children enrolled were collected preferably from the vaccination card, and alternatively, from parent recall (when the card was not presented or available). Furthermore, data on socio-demographic characteristics of the child and parents together with household characteristics and possessions were collected by interviewing the parent or guardian of each child.

In this study, we used the number of people living in one room, type of water source, type of toilet, possession of a television, a car, a motorbike, a telephone, a fridge, type of cooking fuel, and type of floor materials for the household wealth index construction.

### Data management and data analysis

The database was cleaned by visually checking for data consistency in MS-Excel 2019. Data analysis was done with R version 4.1.0 (2021–05-18).

Generated with principal components analysis (PCA), the wealth index placed individual households on a continuous scale of relative wealth. In the same manner as calculated in DHS, we separated all interviewed households into five wealth quintiles to assess the association of wealth with full immunisation status.

The immunisation coverage was calculated as a proportion with the corresponding 95% confidence interval (CI), stratified by vaccine doses. The proportion of fully immunised children aged 12–59 months was also calculated as a percentage with its 95% CI. A child was said to be fully immunised in this study if he/she had received the following vaccines based on vaccination card or parent recall: one dose BCG (Bacille Calmette Guérin), 4 doses of OPV (Oral Polio Vaccine), 3 doses of pentavalent vaccine, 3 doses of PCV-13, 2 doses of rotavirus vaccine, one dose of measles/rubella vaccine and one dose of the yellow fever vaccine. We did not include the inactivated polio vaccine(IPV) because it was recently introduced in the EPI in Cameroon. On the other hand, a child was said to be zero-dose if he or she had never received any dose of routine vaccines. To evaluate the effect of household wealth level and other factors on full immunisation and zero-dose in children, each covariate was run using chi2, with fully immunised (yes/no) and zero-dose(yes/no) as the main outcomes respectively. In the assessment of factors associated with full immunization, any covariate having a *p*-value < 0.35, we further included in the multiple logistic regression model to obtain the Adjusted Odds Ratio (AOR) and Adjusted *p*-value (A*p*-value). On the other hand, only predictor variable having a *p*-value < 0.20 were included in the multiple logistic regression model for the assessment of factors associated with zero-dose. Without any backward or forward selection, all eligible variables for multiple logistic regression were maintained in each model. Based on previous publications, there is no consensus on the cut-off *p*-value to use in the selection of variables for multivariate analysis model [18, 19]. We use 0.35 *p*-value cut-off to select variables for the model with full immunization as outcome based on the recommendation made by Chowdhury et al*.* [20] to increase the chance of covariates to be included in the model because the outcome variable is a composite variable including several vaccines doses. We believed that because of the composite nature of the outcome, association with single variable might less pronounced than in multivariate analysis. On the other hand, a cut-off of 0.20 *p*-value was use for select variable for the model with zero-dose as outcome because the outcome is straight forward [20]. For two models, household wealth level and level of education of mother were included irrespective of their *p*-values in univariate analysis because these factors have been reported by several studies to be associated with vaccination coverage. For these models, the threshold of significance was fixed at a *p*-value < 0.05.

## Results

### Sample description

In total, 529 households were included in the study, with 273 children aged 12–59 months enrolled. Among the participants, 142(52%) were girls, 190(69.6%) aged 24–59 months, and 83(30.4%) aged 12–23 months. Vaccination card retention was 16.8% (46): 30.1% (25) in children aged 12–23 months and 11.1%(16) in children aged 24–59 months. The parent’s recall was the source of information on immunisation status of 83.2% participants.

### Vaccination coverage for the various vaccines

Table 1 presents the results of immunisation coverage per vaccine dose and age group. In all, 37% of children 12–59 months were fully vaccinated according to the national routine immunisation calendar for children 0–11 months. The vaccination coverage for BCG was 78% and 67% for measles/rubella vaccine (1st dose). The proportion of zero dose in our sample was 16% of children 12–59 months. Surprisingly, the coverage of Oral Polio Vaccine (OPV-0) which is normally administered with BCG at the same time was 53% in children 12–59 months. Besides, the vaccination coverage for vaccines administered as multiple doses was incredibly low compared to BCG, measles and yellow fever vaccines which are single dose vaccines.

**Table 1:** Immunisation coverage in children aged 12–59 months

Antigens	Vaccination coverage for the various age groups					
	**Children 12–23 months (*N* = 83)**		**Children 24–59 months (*N* = 190)**		**Children 12–59 months (*N* = 273)**	
	***Freq***	***Coverage[95%CI] (%)***	***Freq***	***Coverage[95%CI] (%)***	***Freq***	***Coverage[95%CI] (%)***
BCG	69	83.13[72.98, 90.15]	145	76.32[69.51, 82.04]	214	78.39[72.93, 83.02]
Polio0	49	59.04[47.69, 69.72]	98	51.58[44.23, 58.87]	147	53.84[47.74, 59.84]
Polio1	44	53.01[41.80, 63.94]	94	49.74[42.19, 56.78]	138	50.55[44.47, 56.61]
DPT + HepB + Hib1	45	54.22[42.96, 65.08]	100	52.63[54.29, 59.86]	145	53.11[47.01, 59.13]
Pneumo1	43	51.81[40.64, 62.81]	96	50.53[43.22, 57.81]	139	50.92[44.83, 56.97]
Rotavirus vaccine1	40	48.19[37.19, 59.36]	87	45.79[38.60, 53.15]	127	46.52[40.51, 53.63]
Polio2	36	43.37[32.68, 54.69]	73	38.42[31.55, 45.76]	109	39.93[34.12, 46.02]
DPT + HepB + Hib2	40	48.19[37.19, 59.36]	93	48.95[41.67, 56.26]	133	48.72[42.67, 54.80]
Pneumo2	38	45.78[34.92, 57.04]	89	46.84[39.62, 54.19]	127	46.52[40.51, 52.63]
Rotavirus vaccine2	37	44.58[33.80, 55.86]	77	40.53[33.55, 47.89]	114	41.76[35.89, 47.87]
Polio3	25	30.12[20.79, 41.32]	41	21.58[16.09, 28.24]	66	24.18[19.31, 29.78]
DPT + HepB + Hib3	33	39.76[29.36, 51.12]	67	35.26[28.58, 42.56]	100	36.63[30.96, 42.68]
Pneumo3	32	38.55[28.26, 49.91]	65	34.21[27.59, 41.48]	97	35.53[29.92, 41.56]
Measles/rubeola vaccine	56	67.47[56.19, 77.11]	129	67.89[60.69, 74.37]	185	67.77[61.82, 73.20]
Yellow Fever vaccine	55	66.27[54.96, 76.05]	129	67.89[60.69, 74.37]	184	67.40[61.44, 72.86]
Fully vaccinated	32	38.55[28.07, 49.88]	69	36.32[29.48, 43.59]	101	37.00[31.26, 43.02]
Zero dose	**10**	**12.05[5.92, 21.04]**	**35**	**18.42[13.18, 34.68]**	**45**	**16.48[12.28, 21.43]**

### Factors associated with full vaccination and zero vaccine dose in children

Table 2 presents factors associated with full vaccination in children aged 12–59 months. The higher level of education of the mother and child living in the same household with the father were significantly associated with higher odds of full vaccination in children. On the other hand, Table 3 presents factors associated with zero vaccine dose in the study participants. Higher level of education of the mother, child living in the same household with the father, higher household wealth index (3^rd^ – 5^th^ quintiles), and the guardian being one of the biological parents of the child were significantly associated with low odds of zero-dose.

**Table 2:** Factors associated with full immunisation in children aged 12–59 months

**Factor**	Bivariate analysis		Multivariate Logistic Regression	
	OR [95% CI]	*p*-value	AOR [95% CI]	A*p*-value
**Gender**				
*Female/Male*	0.62 [0.38, 1.02]	0.05867	0.60 [0.35, 1.04]	0.067
**Age group**				
*24-59 months/12-23 months*	0.91[0.53, 1.55]	0.7245		
**Structure of birth**				
*Health facility/ Community*	1.79 [0.35, 9.04]	0.4756		
**Birth order**				
*Second or higher/ First order*	0.85 [0.49, 1.48]	0.5725		
**Place of residence**				
*Urban area/ Rural area*	2.10 [0.43, 10.31]	0.3505		
**Level of education of Father**				
*Primary school/ Never schooled*	0.78 [0.20, 3.11]	0.7269	0.55 [0.11, 2.77]	0.4707
*Secondary school/ Never schooled*	1.56 [0.45, 5.36]	0.4782	0.92 [0.20,4.34]	0.9168
*High school/ Never schooled*	3.09 [1.02, 9.37]	0.0456*	1.50 [0.34, 6.59]	0.5906
*Higher education/ Never schooled*	2.08 [0.70, 6.17]	0.1851	0.94 [0.21, 4.13]	0.9311
**Age of the father**				
*30 years or older/* < *30 years*	0.91 [0.56, 1.49]	0.72086		
**Child residing with Father?**				
*Yes/No*	2.20 [1.27, 3.82]	0.0044*	2.11 [1.07, 4.16]	0.0305*
**Is the father alive?**				
*Yes/No*	1.72 [0.24, 12.38]	0.58735		
**Employment status of father**				
*Employed/ Unemployed*	1.8948 [0.93, 3.84]	0.0736	1.36 [0.59, 3.15]	0.4673
**Level of education of Mother**				
*Primary school/Never schooled*	3.59 [1.15, 11.19]	0.0275*	5.05 [1.29, 19.76]	0.0200*
*Secondary school/ Never schooled*	1.24 [0.42, 3.62]	0.7000	1.43 [0.38, 5.43]	0.5972
*High school/ Never schooled*	3.92 [1.51, 10.15]	0.0049*	3.68 [1.06, 12.79]	0.0400*
*Higher education/ Never schooled*	6.01 [2.19, 16.55]	0.0005*	8.25 [2.19, 31.16]	0.0018*
**Age of the mother**				
*30 years or older/* < *30 years*	0.83 [0.50, 1.38]	0.4633		
**Employment status of mother**				
*Employed/ Unemployed*	0.60 [0.36, 0.98]	0.0405*	0.69 [0.39, 1.23]	0.1974
**Marital status of the mother**				
*Union(marriage)/ Single*	1.36 [0.79, 2.33]	0.2600		
**Household wealth index level**				
*3*^*rd*^*-5*^*th*^* quintiles/1*^*st*^* and 2*^*nd*^* quintiles*	1.15 [0.68, 1.95]	0.5891	0.90 [0.49, 1.65]	0.7348
**Relationship with guardian**				
*Mother/ Neither father nor mother*	1.50 [0.86, 2.61]	0.1547	1.47 [0.71, 3.06]	0.2972
*Father/ Neither father nor mother*	1.57 [0.60, 4.13]	0.3600	1.11 [0.36, 3.43]	0.8524
**Vaccination card**				
***Present/absent***	0.86 [0.44, 1.65]	0.6473		

NB: only covariates with a *p*-value < 0.35 in the bivariate analysis were included in the multivariate analysis for adjustment. *p-value ≤ 0.05 (below the threshold of significance)

**Table 3:** Factors associated with zero- dose in children aged 12–59 months

**Factor**	Bivariate analysis		Multivariate Logistic Regression	
	OR [95% CI]	*p*-value	AOR [95% CI]	A*p*-value
**Gender**				
*Female/Male*	0.96[0.50, 1.81]	0.8944		
**Age group**				
*24-59 months/12-23 months*	1.65[0.77, 3.51]	0.1917	2.76[).99, 7.63]	0.0512
**Structure of birth**				
*Health facility/ Community*	1.18[0.04, 0.76]	0.0095*	0.00[0.00, 1.0Exp12]	0.9570
**Birth order**				
*Second or higher/ First order*	1.34[0.62, 2.86]	0.4537		
**Place of residence**				
*Urban area/ Rural area*	0.38[0.09, 1.57]	0.1659	> 10.00[0.00, 1.0Exp12]	0.9620
**Level of education of Father**				
*Primary school/ Never schooled*	0.26[0.05, 1.50]	0.1321	0.19[0.02, 1.89]	0.1568
*Secondary school/ Never schooled*	0.33[0.07, 1.51]	0.1514	0.94[0.10, 8.95]	0.9553
*High school/ Never schooled*	0.17[0.04, 0.77]	0.0212*	0.27[0.02, 3.04]	0.2921
*Higher education/ Never schooled*	0.14[0.03, 0.65]	0.0115*	0.30[0.03, 3.33]	0.3248
**Age of the father**				
*30 years or older/* < *30 years*	0.33[0.16, 0.66]	0.0012*	1.51[0.50, 4.52]	0.4618
**Child residing with Father?**				
*Yes/No*	0.28[0.14, 0.54]	0.0001*	0.22[0.06, 0.83]	0.0253*
**Is the father alive?**				
*Yes/No*	1.70[0.17, 16.77]	0.6437		
**Employment status of father**				
*Employed/ Unemployed*	0.49[0.22, 1.10]	0.0783	0.87[0.26, 2.95]	0.8280
**Level of education of Mother**				
*Primary school/Never schooled*	0.34[0.07, 1.74]	0.1938	0.07[0.01, 0.74]	0.0271*
*Secondary school/ Never schooled*	0.57[0.18, 1.80]	0.3365	0.28[0.04, 1.84]	0.1842
*High school/ Never schooled*	0.47[0.16, 1.41]	0.1773	0.55[0.09, 3.44]	0.5214
*Higher education/ Never schooled*	0.18[0.04, 0.93]	0.0406*	0.10[0.01, 0.92]	0.0419*
**Age of the mother**				
*30 years or older/* < *30 years*	1.08[0.56, 2.08]	0.8164		
**Employment status of mother**				
*Employed/ Unemployed*	2.59[1.29, 5.20]	0.0059*	1.69[0.61, 4.67]	0.3153
**Marital status of the mother**				
*Union(marriage)/ Single*	0.42[0.22, 0.80]	0.0073*	1.10[0.33, 3.66]	0.8761
**Household wealth index level**				
*3*^*rd*^*-5*^*th*^* quintiles/1*^*st*^* and 2*^*nd*^* quintiles*	0.41[0.22, 0.79]	0.0069*	0.25[0.10, 0.67]	0.0053*
**Relationship with guardian**				
*Mother/ Neither father nor mother*	0.11[0.05, 0.24]	< 0.0001*	0.05[0.02, 0.18]	< 0.0001*
*Father/ Neither father nor mother*	0.08[0.01, 0.58]	0.0134*	0.10[0.01, 1.10]	0.0594

NB: only covariates with a *p*-value < 0.20 in the bivariate analysis were included in the multivariate analysis for adjustment. *p-value ≤ 0.05 (below the threshold of significance)

## Discussions

### Summary

This paper aimed to evaluate socio-demographic factors associated with full vaccination and zero-dose for the Expanded Programme on Immunisation (EPI) in Yaounde-Cameroon. The results unveiled that single-dose vaccines such as BCG and measles vaccine had better coverage compared to multiple-dose vaccines. Also, children whose mother had higher level of education and those living with their fathers in the same household had significantly higher odds of being fully vaccinated and lower odds of zero-dose of EPI vaccines. In addition, children whose guardians were not any of their biological parents, those living in households with level wealth index were significantly more likely to be zero-dose.

### Vaccination coverage of different EPI vaccines

The overall aim of the routine EPI in Cameroon is to reduce the morbidity and mortality associated with vaccine-preventable diseases [11]. To achieve this goal, immunisation coverage among the target population must be high to ensure herd immunity and hence collective protection. Therefore, the Cameroon government’s target is to vaccinate at least 80% of the target population at the district level and 90% at the national level [11, 21]. Our findings show that vaccination coverage for EPI vaccines in Yaounde is low below the target of the national programme (< 80%) except for the BCG vaccine with a coverage of 83% in children aged 12–23 months. However, the coverage of BCG vaccine, measles vaccine, yellow fever vaccine, and the proportion of zero dose in children aged 12–23 months are similar to the results of the 2018 DHS in Cameroon [10] and a study in Dschang health district [14]. For the other vaccine types(multiple-dose vaccines) like OPV, Pentavalent vaccine, PCV-13, and proportion of fully vaccinated children, we found a much lesser coverage (approximately 2 times lesser) compared to the results of the 2018 DHS, but much higher than the findings from Foumban, a rural district in West Cameroon [6].

The exact reason the immunisation coverage for multiple-dose vaccines and the proportion of fully vaccinated children in this study are quite different from the results of the DHS 2018 is not known. However, we believe that it could be linked to recall bias since 83.2% of the data collection was based on recall against 47% in DHS 2018 [10]. A study in Dschang reported that immunisation coverage in children was significantly associated with the presence of the vaccination card [14]. Similar studies align with the fact that parent recall was more frequently associated with under-reporting of vaccination status in children [15–17]. The higher vaccination card retention proportion reported in Dschang [33%] [14] and the DHS 2018 [53%] [10] could be at the origin of the differences in these findings as our study reports only a card retention proportion of 16.8%. If this hypothesis is true, it shows that the recall bias for routine immunisation depends on the vaccine in question and affects multiple-dose vaccines more than the single-dose vaccines. However, this needs further investigation. Another explanation to the differences in immunisation coverage observed in this study compared to DHS 2018 may be related to age groups of the participants. In this study, close to 70% of participants were above 23 months, while previous studies targeted children aged 12–23 months. This age difference in participants might have given more room for recall bias in our study. Besides, the 2018 DHS was a national representative survey including participants across all the 10 regions, Douala and Yaounde inclusive, while our study was conducted only in the 6 health districts of Yaounde.

Surprisingly, the coverage of Oral Polio Vaccine (OPV-0) which is normally administered with BCG at the same time was 53% in children 12–59 months against 78% coverage for BCG. The exact reason for this difference is not known to us. However, we believe this is associated with recall bias. While OPV is expected to less affected by recall bias because it is administered orally, we think that the very low vaccination card retention in our study population might have compounded the effect of recall bias. On the other hand, the presence of a scar facilitates proof of BCG vaccination.

Based on the results of this study, the vaccination coverage in Yaounde is sub-optimal and needs more efforts to attain the target of 80% per district per vaccine as stipulated by the national guidelines for the EPI [11]. When compared with the results from previous surveys, it suggests that there is a high disparity of vaccine uptake in different districts in Cameroon affecting OPV, PCV-13, Pentavalent vaccine, and rotavirus vaccine. Understanding the causes of low vaccine uptake can help find a lasting solution to the problem of immunisation uptake in Yaounde and Cameroon as a whole.

### Factors associated with full vaccination and zero-dose

The findings of this study revealed that children of highly educated mothers and those sharing the same households with their biological fathers were more likely to be fully vaccinated with the EPI vaccines. Though no meaningful relationship was found between full vaccination and household wealth level, household wealth showed a significant association with zero-dose. Another interesting finding was that home births were significantly associated with zero-dose in the bivariate analysis. However, this could not yield interpretable results in multivariate analysis due to extremely small number of community births. In accord with our findings, other studies in Ethiopia and Cameroon reported that children of mothers with a higher level of education were likely to be fully vaccinated [6, 14, 22, 23]. This suggests that ensuring proper education of a girl child would be a long terms effective strategy to improve immunisation update in the future.

Several studies have reported a relationship between full immunisation in children and their household wealth level, where it was demonstrated that children in households with higher wealth levels were more likely to be fully immunised [22, 23]. However, to the best of our search, no study has evaluated the relationship between zero-dose and household wealth. Both full vaccination proportion and proportion of zero-dose are indicators of vaccination access. Given that EPI services in Cameroon are free of charge, the disparity of immunisation coverage among children in households of different wealth classes indicates that, making EPI vaccines free of charge is not sufficient to ensure equitable access to vaccines. Though the reason for the differences in immunisation coverage between the rich and the poor is not known with certainty, we believe that this might be caused by some associated costs like transport fair to access health facility, purchase of vaccination card, access to information (through radio, TV, newspapers, etc.), and differences in the level of education [6, 22, 23]. Besides, previous studies reported other factors such as the source of information on vaccination status, coming to a health facility within one month of birth for postnatal care, and the long distance to reach the vaccination centre [6, 22, 23]. Notwithstanding, the relationship between immunisation and household wealth level is persistently similar across studies and contexts [24, 25].

### Study limitations

These results could be potentially exposed to recall bias because more than 83% of the children did not have any proof of immunisation (vaccination card) and hence assessment was based only on parents recall. Besides, including children aged 12–59 months compounded more the potential of recall bias. Also, being a cross-sectional study, the findings are valid for Yaounde(the study area) and cannot be inferred to Cameroon as a whole.

However, this study design was the best method to conduct such studies because most of the factors assessed can change with time and become difficult to evaluate with prospective studies. The findings can be used to guide EPI activities in Yaounde and it can equally be used to generate hypothesis for further investigations.

## Conclusions and recommendations

Approximately 37% of children aged 12–59 months in Yaounde are fully vaccinated according to the national EPI calendar for children. The immunisation coverage of different vaccine doses varies widely with the vaccine (BCG, measles, yellow fever, reporting coverage above 60% and other vaccines less). This immunisation coverage is still far below the national target of 90%. Also, factors positively associated with full vaccination in children include higher level of education of the mother and living in the same household as the father. In the same way, living in a richer household (3^rd^-5^th^ household wealth index quintiles), having an educated mother and the guardian being the biological parent of the child had significant negative association with zero-dose in children aged 12–59 months in Yaounde.

As reported in several studies, disparity persists in the uptake of vaccines between the poor and the rich, despite the free nature of vaccination services in Cameroon. Exploring the root causes of the intermediary factors responsible for this disproportionate vaccine uptake between the poor and the rich would be a valuable topic for further research. Further study is recommended to assess the effect of home births on zero-dose for EPI vaccine in children. On the other hand, immunisation program managers need to rethink a better approach to reach the poor with immunisation services.

## Data Availability

The datasets used and/or analysed during the current study are available from the corresponding author on reasonable request.

## Abbreviations

AOR: Adjusted Odds ratio
Ap-value: Adjusted *p*-value
BCG: Calmette-Guérin Bacillus (vaccine)
95% CI: 95 Percent Confidence interval
CMA: Sub-divisional Hospital (Centre Medical d’Arrondissement)
DHS: Demographic Health Survey
DPT-HepB + Hib: Diphtheria, Pertussis, Tetanus and Hepatitis B + Haemophilus Influenzae type b
EPI: Expanded Program of Immunization
GAVI: Global Alliance for Vaccination and Immunization
Hib: Haemophilus Influenzae type b
Hepb: Hepatitis B vaccine
IPV: Inactivated Polio Vaccine
MR: Measles and Rubella vaccine
MoPH: Ministry of Public Health
MoH: Ministry of Health
INS: National Institute of Statistics
OR: Odds ratio
OPV: Oral Polio Vaccine
PCV-13: Pneumococcal Conjugate Vaccine 13
Penta (pentavalent vaccine): Diphtheria, Pertussis, Tetanus and Hepatitis B + Haemophilus Influenzae type b
PCA: Principal Component Analysis
PPS: Probability Proportionate to Size
UNICEF: United Nations Children’s Fund
WI: Wealth index
WHO: World Health Organization
YF: Yellow Fever
